# Zika Virus, Chikungunya Virus, and Dengue Virus in Cerebrospinal Fluid from Adults with Neurological Manifestations, Guayaquil, Ecuador

**DOI:** 10.3389/fmicb.2017.00042

**Published:** 2017-01-24

**Authors:** Nathalie Acevedo, Jesse Waggoner, Michelle Rodriguez, Lissette Rivera, José Landivar, Benjamin Pinsky, Hector Zambrano

**Affiliations:** ^1^Laboratorio de Biología Molecular, Hospital Luis VernazaGuayaquil, Ecuador; ^2^Department of Medicine, Division of Infectious Diseases, Emory University School of Medicine, AtlantaGA, USA; ^3^Departamento de Medicina Interna, Hospital Luis VernazaGuayaquil, Ecuador; ^4^Department of Pathology, Stanford University School of Medicine, StanfordCA, USA; ^5^Department of Medicine, Division of Infectious Diseases and Geographic Medicine, Stanford University School of Medicine, StanfordCA, USA

**Keywords:** Zika virus, Guillain–Barre syndrome, meningitis, chikungunya virus, dengue virus, cerebrospinal fluid, molecular diagnosis

## Abstract

Zika virus (ZIKV), chikungunya virus (CHIKV), and dengue virus (DENV) have been associated with clinical presentations that involve acute neurological complaints. In the current study, we identified ZIKV, CHIKV, and DENV in cerebrospinal fluid (CSF) samples from patients admitted to the Hospital Luis Vernaza (Guayaquil, Ecuador) to the Emergency Room or the Intensive Care Unit, with neurological symptoms and/or concern for acute arboviral infections. Viral RNA from one or more virus was detected in 12/16 patients. Six patients were diagnosed with meningitis or encephalitis, three with Guillain–Barré Syndrome, and one with CNS vasculitis. Two additional patients had a systemic febrile illness including headache that prompted testing of CSF. Two patients, who were diagnosed with encephalitis and meningoencephalitis, died during their hospitalizations. These cases demonstrate the breadth and significance of neurological manifestations associated with ZIKV, CHIKV, and DENV infections.

## Introduction

Zika virus (ZIKV) is an arthropod-borne virus (arbovirus) of the genus *Flavivirus* (family *Flaviviridae*) that is transmitted by the same mosquito vectors as chikungunya virus (CHIKV) and dengue virus (DENV; Waggoner and Pinsky, 2016). Amidst the current outbreak in the Americas, ZIKV has been associated with severe neurological manifestations in adults, including Guillain–Barré syndrome (GBS; Cao-Lormeau et al., 2016; Dirlikov et al., 2016; do Rosario et al., 2016; Dos Santos et al., 2016), acute myelitis (Mecharles et al., 2016), and encephalitis (Carteaux et al., 2016; Soares et al., 2016). GBS has been the best studied neurological presentation in adults, and the association between recent ZIKV infection and GBS has been principally demonstrated in studies that have relied on serologic testing for ZIKV diagnosis (Cao-Lormeau et al., 2016; Dos Santos et al., 2016). This may be due to difficulties in diagnosing acute ZIKV infections in adults, as detection of ZIKV in CSF has been infrequently reported (Parra et al., 2016; Rozé et al., 2016; Siu et al., 2016; Zambrano et al., 2016) and serologic testing may be difficult to interpret in DENV endemic regions (Waggoner and Pinsky, 2016).

Zika virus, CHIKV, and DENV have co-circulated in many regions of the Americas over the past year. While the neurological complications of ZIKV infection have been the cause of much concern during this outbreak, less attention has been paid to the neurological manifestations associated with acute or recent CHIKV and/or DENV infections (Solomon et al., 2000; Gerardin et al., 2016). Clinical diagnosis cannot reliably distinguish symptomatic infections caused by these viruses (Waggoner et al., 2016b), which complicates the interpretation of results from studies based on reported Zika cases (Dos Santos et al., 2016) or patients with compatible symptoms (Parra et al., 2016). Additionally, co-infections between these viruses may be common in certain settings (Villamil-Gomez et al., 2016; Waggoner et al., 2016b; Zambrano et al., 2016), and the detection of co-infections in cases of CNS disease has not been well described (Zambrano et al., 2016).

The first cases of ZIKV infection in Ecuador were reported in January 2016 in two returned travelers. Shortly thereafter, autochthonous transmission was documented in the country. CHIKV was introduced into Ecuador in 2014, and DENV has been endemic in Ecuador since 1988. Guayaquil, located on the Pacific Ocean, is the largest city in Ecuador, and has a high incidence of arboviral infections. During 2016, ZIKV, CHIKV, and DENV co-circulated in Guayaquil. In the current study, we sought to determine the incidence of ZIKV, CHIKV, and/or DENV detection in cerebrospinal fluid (CSF) from adult patients admitted with neurological symptoms to the Hospital Luis Vernaza, the largest medical center in Guayaquil.

## Materials and Methods

### Clinical Samples

A convenience set of CSF samples from 16 patients was tested for this study. Patients had been admitted to the Hospital Luis Vernaza in Guayaquil, Ecuador and had CSF collected as part of routine care. Patients were admitted from February 1 to August 31, 2016 and were initially evaluated by a neurologist or hospital staff physician. If lumbar puncture was warranted, the procedure was performed and CSF was sent to the Molecular Biology Laboratory and the Hospital Central Laboratory. Following the performance of testing requested by care providers, remaining CSF was stored at -20°C until RNA extraction could be performed. When possible, urine and blood samples collected on the day of CSF collection were also tested.

In addition to molecular testing for ZIKV, CHIKV, and DENV (described below), CSF samples were evaluated by the following methods: Gram stain, bacterial culture (up to 48 h if no growth was observed), Ziehl–Neelsen stain, and India ink stain. CSF PCRs were also performed for herpesviruses [HSV 1, 2, and 6; cytomegalovirus; Epstein–Barr virus (EBV); and varicella zoster virus, all from DiaPro, Milan, Italy], toxoplasma gondii (DiaPro, Milan, Italy), enterovirus and tuberculosis (Cepheid, Sunnyvale, California) according manufacturer recommendations. Clinical data and the results of additional laboratory tests (e.g., blood cell counts, biochemical, and microbiological results from CSF), electromyography, and radiographic studies were obtained from the medical record. Patients provided written informed consent for diagnostic procedures and laboratory testing recommended by their care providers. For patients with altered cognition, consent was obtained from a surrogate decision maker. The study protocol was reviewed and approved by the Comité de Investigación at the Hospital Luis Vernaza.

### Sample Processing and Arbovirus Testing

RNA was extracted from140 μl CSF for all patients using the QIAamp Viral RNA Mini kit (Qiagen, Hilden, Germany) and a 60-μL elution volume, according to the manufacturer’s instructions. RNA was also extracted from serum (*n* = 4) and urine (*n* = 3) samples, using the same protocol and specimen volumes. RNA was stored at -20°C until testing.

All samples were tested for ZIKV, CHIKV, and DENV using the ZCD assay. This is an internally controlled, multiplex real-time reverse transcription PCR (rRT-PCR), which was performed on a Cobas Z 480 instrument (Roche Diagnostics) using the users defined format, as described previously (Waggoner et al., 2016a; Zambrano et al., 2016). ZIKV, CHIKV, and DENV are detected in separate channels of the Cobas Z, and an assay for RNase P detection serves as a heterologous, intrinsic internal control. A sample was considered positive for a given target (any virus or RNase P) if an exponential curve crossed the instrument-defined threshold in the appropriate channel prior to or at cycle 40. Samples negative for all three viruses and with a positive result for RNase P were considered negative. Each run of the ZCD assay included a no-template control (water), a negative control (positive for RNase P but negative for any pathogen), and positive controls for ZIKV, CHIKV, and DENV.

## Results

Cerebrospinal fluid samples from 16 patients were included in this study (**Table 1**). The mean patient age was 42.1 years (sd 17.4) and 10 patients (62.5%) were male. Results of testing using the ZCD assay are shown in **Table 1**. Twelve samples (75.0%) tested positive for one or more viruses: ZIKV was detected in nine patients, CHIKV in 11, and DENV in 5. Three patients had evidence of a mono-infection, and nine patients had evidence of a co-infection with two (*n* = 5) or all three viruses (*n* = 4; **Table 1**). Viral RNA was detected as late as 14 days post-symptom onset. Four individuals tested negative in the ZCD assay but had detectable RNase P, indicating sufficient nucleic acid extraction and the absence of PCR inhibitors.

**Table 1:** Demographic information and ZCD assay results from CSF for 16 patients.

Patient	Age (years)	Gender	Days post-symptom onset	ZCD assay results			
				
				ZIKV	CHIKV	DENV	RNase P
1	18	Male	5	+	+	+	+
2	16	Male	3	-	-	-	+
3	23	Male	1	+	+	+	+
4	54	Female	6	+	+	-	+
5	44	Male	9	+	+	-	+
6	25	Female	14	+	+	+	+
7	47	Male	1	+	+	-	+
8	28	Female	6	+	+	-	+
9	65	Female	1	-	-	-	+
10	48	Male	14	-	+	+	+
11	48	Male	15	-	-	-	+
12	60	Female	5	-	+	-	+
13	53	Female	14	-	+	-	+
14	62	Male	1	+	+	+	+
15	21	Male	8	+	-	-	+
16	62	Male	30	-	-	-	+


Clinical information on the 12 positive patients is shown in **Table 2**. Six patients were diagnosed with meningitis or encephalitis, three patients had GBS, and one patient was diagnosed with CSF vasculitis. In two additional cases, patients had a systemic febrile illness related to a ZIKV-CHIKV co-infection or CHIKV mono-infection. Two patients with meningitis had AIDS and were also diagnosed with toxoplasmosis and cryptococcosis, respectively, during their admissions. Patients 3 and 12 were both admitted to the intensive care unit with encephalitis and meningoencephalitis, respectively, and died during their hospitalization.

**Table 2:** Clinical information for 12 patients with positive ZCD assay results from CSF.

Patient	Signs and symptoms	Diagnosis	Additional positive results
1	Upper respiratory tract infection, followed by asthenia, leg paresthesias, weakness, dysarthria, decreased muscular strength in legs and left arm (3/5), hyporeflexia, hyporeactive pupils.	Guillain–Barré syndrome. Symmetric motor polyneuropathy	None
3	Encephalopathy, asthenia, oliguria	Encephalitis	None
4	Fever, headache, and lumbar back pain; followed by paresthesias in her hands, feet, and face; left facial paralysis; and dysarthria.	Guillain–Barré syndrome. Motor and sensory axonal neuropathy	None
5	Nosocomial fever of unknown origin, arthralgia	Fever secondary to ZIKV-CHIKV co-infection	None
6	Fever, nausea, vomiting and diarrhea; followed by generalized tonic-clonic seizure, headache, ptosis of the eyelids, neck stiffness.	Meningitis	None
7	Altered wakefulness, generalized tremors and walking difficulties. Fever and tonic-clonic seizure.	Encephalitis	None
8	Fever, generalized weakness, asthenia, anorexia, headache, neck stiffness	Meningitis	HIV/AIDS Toxoplasmosis detected by CSF PCR
10	Fever, vomiting, diarrhea and headache	Meningitis	HIV/AIDS *Cryptococcus* detected by India Ink and culture
12	Cold symptoms followed by fever, chills, headache, neck stiffness, nausea, vomiting, sensory impairment, photophobia	Meningoencephalitis	EBV detected by CSF PCR
13	Polyarthralgia, fever and headache	Chikungunya fever	Self-reported history of chikungunya
14	Fever, sweating, paraplegia, arreflexia, dyspnea, decreased muscular strength in arms	Guillain–Barré syndrome	None
15	Blurred vision in left eye followed by blindness, dysphasia, weakness and decreased muscular strength in legs and arms, lack of sphincter control.	Cerebral vasculitis	None


Cerebrospinal fluid findings for eight patients who had ZIKV, CHIKV, and/or DENV detected in CSF without another potential cause for their symptoms are shown in **Table 3**. Five of eight patients had an elevated white blood cell (WBC) count in the CSF (≥5 cells/μL), and patients 6 and 13 had marked elevations to 245 and 142 cells/μL, respectively. Three patients with CSF leukocytosis had a predominance of mononuclear cells. Patient 7, who presented 1 day post-symptom onset, had a neutrophil predominance. Five patients had elevated CSF protein (>40 mg/dl), including all three patients with normal CSF WBC counts. Mild elevations in CSF lactate dehydrogenase (LDH) and decreases in glucose were seen, together, in patients 7 and 13. While both patients had CHIKV infections, these changes did not correlate with clinical presentation or disease severity.

**Table 3:** Cytological analysis for patients with ZIKV, CHIKV, and/or DENV detected from CSF without another potential cause for their presentation.

Patient^∗^	Leukocyte count (per μl)	Neutrophils (%)	Mononuclear cells (%)	Glucose (mg/dl)	Protein (mg/dl)	LDH (U/l)
1	13	38.5	61.5	55	33.3	11
3	5	40	60	79.1	50.9	27
4	3	66.7	33.3	57.1	120.2	16
6	245	18	82	56.1	20.3	25
7	6	83.3	16.7	36.6	76.0	43
13	142	4.9	95.1	31.3	62.0	66
14	2	50	50	68	278	22
15	1	0	100	59.4	54	22


*^∗^For patient 5, there was clinical concern for a post-operative infection, though no growth was observed in bacterial cultures.*

In four patients (1, 3, 4, and 12), serum and/or urine samples were available from the same day as the CSF sample. Results from CSF and serum were concordant for 10/12 (83.3%) possible comparisons. In patient 3, DENV was detected in CSF and not serum; in patient 12, ZIKV was detected in serum (and urine) but not in CSF. Patient 4 had concordant results in each specimen type (ZIKV-CHIKV co-infection). Finally in Patient 1, urine tested negative for DENV and CHIKV, though ZIKV, CHIKV, and DENV were all detected in serum and CSF.

### Radiographic Findings and Electromyography Testing

Electromyography findings in patients diagnosed with GBS were consistent with the following: motor axonal polyneuropathy (Patient 1); motor and sensory axonal neuropathy and secondary acute demyelination (Patient 4); demyelinating polyneuropathy and secondary axonal damage (Patient 14). Patient 1 had a brain MRI performed, which was normal.

Three additional patients had abnormalities noted on CNS imaging. Patient 3 had a head computed tomography scan showing symmetric alterations in the lenticular nucleus that were confirmed by magnetic resonance angiography as ischemic alterations of vasculitic origin affecting the lenticular nucleus, periventricular thalamic nucleus and the periaqueductal gray matter. Patient 7 had discrete changes detected in the lenticular nucleus by MRI (T2-flair images) as well as electroencephalogram findings consistent with cortical dysfunction. Patient 15 had multiple abnormalities on brain MRI affecting the periventricular regions, the protuberance and the cerebral peduncle. These did not extend to contiguous structures, and findings were most consistent with cerebral vasculitis.

## Discussion

In the current paper, we present 16 patients who were admitted to a single center in Guayaquil, Ecuador, with neurological symptoms and/or concern for arboviral illness. We identified twelve individuals with detectable RNA from ZIKV, CHIKV, and/or DENV in CSF. Notably, co-infections were identified in CSF relatively frequently (9/12 positive cases). RNA was detectable early in the course of neurological symptoms, which is consistent with a recent report describing detection of ZIKV RNA in the CSF of a patient with GBS on day 3 (Siu et al., 2016).

Recent reports describing GBS in the setting of ZIKV infections have principally relied on serologic testing and/or clinical symptoms for the detection of Zika cases. In a report by Parra et al. (2016), 66/68 patients with GBS in Colombia had symptoms of a recent ZIKV infection. Of 42 patients tested by RT-PCR, only 17 (25% of the total population) were positive for ZIKV, including 16 urine samples and 3 CSF samples. All patients tested negative for DENV by RT-PCR (Parra et al., 2016). In our experience, clinical symptoms do not accurately differentiate patients with ZIKV, CHIKV, and/or DENV (Waggoner et al., 2016b). Reliance on reported symptoms without diagnostic confirmation may over-emphasize the association between GBS and ZIKV mono-infection, and in our series, the three cases of GBS occurred in the setting of ZIKV co-infections with CHIKV and/or DENV.

It is notable that in the current series, the most severe cases involved patients with encephalitis and meningoencephalitis. Both patients tested positive for CHIKV, including patient 12 who only tested positive for CHIKV, and both patients died in the intensive care unit. A small number of deaths have been reported in the setting of ZIKV infection, though these cases have typically presented with anemia and severe thrombocytopenia, rather than neurological manifestations (Sarmiento-Ospina et al., 2016; Swaminathan et al., 2016).

Increased detection in our case series may have resulted from utilization of a multiplex rRT-PCR with improved sensitivity for ZIKV and DENV detection (Waggoner et al., 2016a) compared to assays that were used in studies referenced here (Cao-Lormeau et al., 2016; Dirlikov et al., 2016; Parra et al., 2016). This facilitates the testing of all samples for ZIKV, CHIKV, and DENV in a single reaction, where testing with separate assays may not be performed following a single positive result. Arbovirus RNA was detected in CSF as late as 14 days post-symptom onset. As this specimen type is rarely tested in the setting of acute infections with these pathogens, the duration of RNA detection in CSF is unknown. However, we do not favor that viral detection in these cases was related to past infections, given the paucity of co-infections reported from CSF to date.

Finally, two patients with HIV and AIDS were identified who had ZIKV-CHIKV and CHIKV-DENV co-infections, respectively. Both patients were severely ill, but the contribution of arboviral infection to the clinical picture, in the setting of documented opportunistic infections, is unclear. Only a single case of ZIKV in an HIV-infected patient has been well documented in the literature (Calvet et al., 2016). For DENV, however, AIDS does not appear to be a risk factor for the development of severe disease (Watt et al., 2003), and it is likely that ZIKV, CHIKV, and DENV were incidentally detected in these two cases. Given the small sample size and observational nature of the current study, further conclusions regarding the impact of co-infections on disease manifestations cannot be made, but this warrants further study in endemic regions.

## Conclusion

Our data demonstrate the breadth of neurological manifestations associated with ZIKV, CHIKV, and/or DENV infections. All three viruses should be considered in the differential diagnosis for patients with new neurological symptoms in endemic areas of the world, and these data further support the use of a multiplex diagnostic for ZIKV, CHIKV, and DENV testing.

## Author Contributions

HZ conceived the investigation and supervised the experimental work and data analyses. LR and JL performed the PCR experiments; JW and BP originally described the ZCD assay and provided the primers and probes for the PCR reactions. NA and MR revised and analyzed the medical records of the patients. NA prepared the database with integrated molecular and clinical data. NA, HZ and JW analyzed the data. NA, JW and HZ wrote the manuscript. BP edited the manuscript. All authors revised and approved the final version.

## Conflict of Interest Statement

The authors declare that the research was conducted in the absence of any commercial or financial relationships that could be construed as a potential conflict of interest.
